# Harnessing Interfacial Field Localization in Hexagonal GaP Mie Resonators

**DOI:** 10.1002/nap2.70085

**Published:** 2026-04-30

**Authors:** Aleksandra A. Kutuzova, Alexey Kuznetsov, Valeriy M. Kondratev, Vladimir V. Fedorov, Ivan S. Mukhin, Aleksandr S. Slavich, Aleksey V. Arsenin, Alexey D. Bolshakov, Mikhail V. Rybin

**Affiliations:** ^1^ School of Physics and Engineering ITMO University Saint Petersburg Russia; ^2^ Moscow Center for Advanced Studies Moscow Russia; ^3^ Alferov University Saint‐Petersburg Russia; ^4^ Peter the Great Saint Petersburg Polytechnic University Saint Petersburg Russia; ^5^ Emerging Technologies Research Center XPANCEO Dubai UAE; ^6^ Faculty of Physics Saint‐Petersburg State University Saint Petersburg Russia; ^7^ Ioffe Institute Saint Petersburg Russia

**Keywords:** gallium phosphide, high index, Mie modes, nanowire

## Abstract

Gallium Phosphide (GaP) is a premier material for integrated nanophotonics, combining prominent second‐order nonlinear properties with near‐perfect lattice matching with silicon for CMOS compatibility. However, leveraging these advantages in scalable bottom‐up architectures requires understanding optical properties of the geometries defined by the fabrication methods. Here, we reveal the formation of a highly confined electromagnetic “hot spot” at the nanowire‐substrate interface in self‐assembled GaP nanowires (NW). This robust interfacial field localization is driven by the fundamental TM11 Mie resonance, which is selectively supported by the hexagonal geometry of the epitaxially grown NW. Through full‐wave simulations and polarization‐resolved dark‐field spectroscopy, we find that the hexagonal cross‐section also suppresses higher‐order modes, yielding a simplified and spectrally clean resonant landscape. Experimental measurements confirm the diameter‐dependent tunability of these modes across the visible spectrum. These findings establish a scalable platform for nanoantennas deterministically concentrating light at the substrate interface, ideal for employing the intrinsic nonlinearity of GaP for frequency conversion and enhancing light–matter interactions for quantum emitters and biosensing.

## Graphical abstract

Сharacterization of Mie modes in self‐assembled GaP nanowires grown by MBE in vertical and horizontal configurations. The hexagonal cross‐section suppresses higher‐order modes while preserving dominant low‐order modes with spectral shifts. Simulations and dark‐field spectroscopy confirm the diameter‐dependent tunability. The Mie modes localize at the interface, enhancing light–matter interaction. These nanowires provide a scalable platform for photonic and quantum devices.

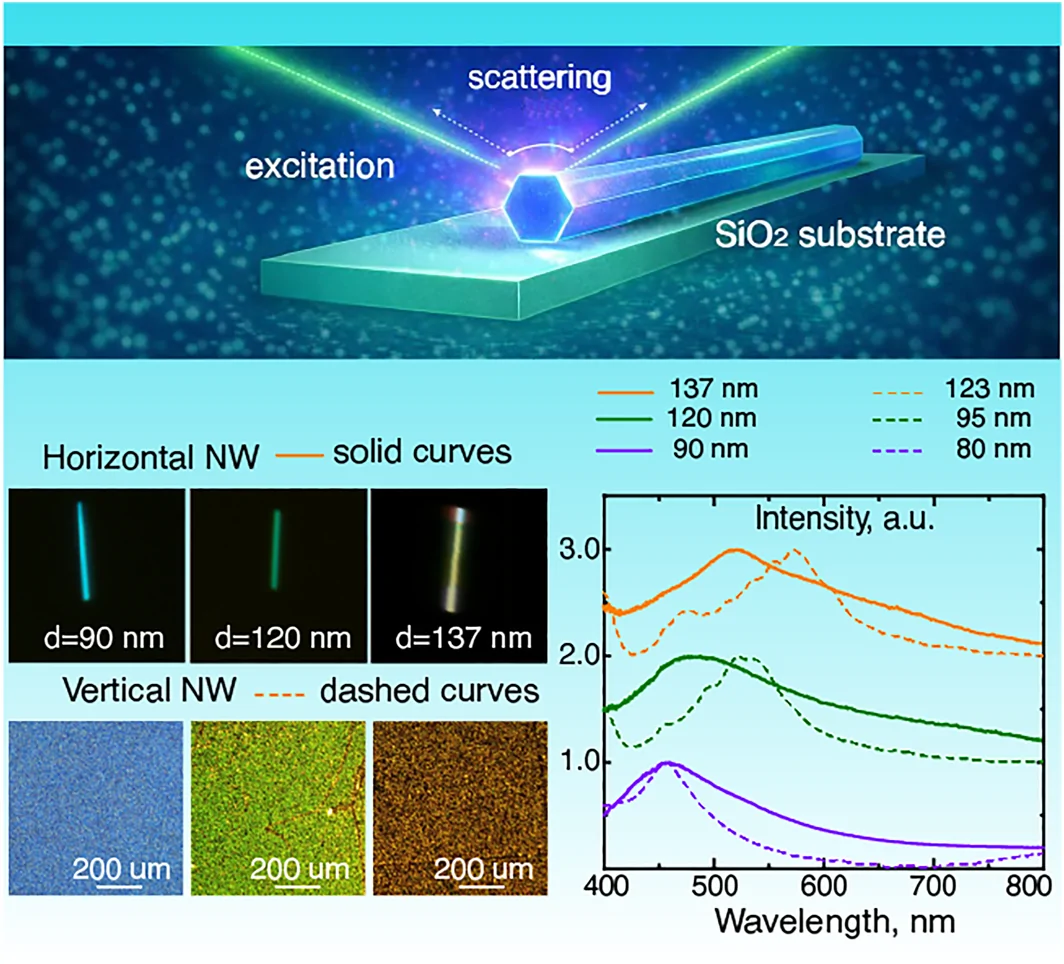

## 1 Introduction

Over the last decade, Mie resonances have been recognized as a key component of nanophotonic applications, also known as Mie‐tronics [1, 2, 3]. Typically, high‐quality (Q) factor Mie resonances are supported in high‐index subwavelength nanoparticles, which confine the electromagnetic energy [4, 5]. The resonant enhancement of the light–matter interaction is used in sensing for signal amplification [6], in photovoltaics to boost the efficiency [7, 8, 9], in imaging to improve the contrast and resolution [10, 11, 12], to name a few. Other applications include efficient light emitters and modulators [13, 14, 15, 16], optical coupling and guiding [17, 18, 19], nonlinear harmonic generation [20, 21], and even telecommunications [22].

Although the Mie problem was mathematically solved a century ago, many issues remain, hindering its practical implementation [23, 24, 25, 26]. There are two major factors that dramatically change the particle response—the high‐index material and the geometrical shape—and both of them are strongly related to the available technology [27]. Moreover, the chosen material defines the possible geometries to a certain extent [28]. Modern nanophotonics is mostly based on silicon, which is CMOS‐compatible and hence cost‐effective [29, 30]. However, silicon exhibits absorption in the visible range, which limits its applications. Other materials such as GaAs or TMDC have strong nonlinearity and anisotropy [31]. Recently, gallium phosphide (GaP) attracted attention as a promising material for nanophotonics due to its indirect bandgap, low absorption from visible to IR wavelengths (0.5–11 μm), and high refractive index of 3.5 [32, 33].

Bottom‐up approaches are much cheaper than top‐down lithographical methods. Epitaxial growth methods allow production of large ensembles of similar GaP nanowires (NW) in a single technological process, with precise control over the NW dimensions and crystalline phase, yielding high‐performance optical resonators that can be arranged in dense arrays without the need for complex post‐fabrication processing. GaP is known for its excellent chemical stability and does not readily form a native oxide layer under ambient conditions, unlike silicon or arsenide‐containing compounds. Owing to this resistance to oxidation, GaP nanowires do not develop unintended core–shell [34, 35] architectures associated with surface oxide formation. NW were shown to enable strong electromagnetic field localization [36] and spectral modulations [37, 38], and facilitate stimulated emission at significantly reduced pump power thresholds [39], which makes them excellent candidates for nanolaser applications. Furthermore, the interplay between their superior scattering properties and strong nonlinear response [40] creates ideal conditions for developing tunable multi‐harmonic light sources. Although we use GaP NW with a hexagonal cross‐section resulting from epitaxial growth as a representative example, our study addresses a broader issue: how the intrinsic hexagonal geometry modifies Mie resonances compared with idealized cylindrical models in realistic scalable NW systems? Moreover, the analysis of the Mie modes in GaP NW accounting for the material, geometry, and growth configuration, is yet to be carried out.

We present a combined theoretical and experimental investigation of Mie resonances in self‐assembled GaP NW, comparing hexagonal geometries (typical for epitaxial NW) with conventional cylindrical ones in both vertically standing and planarized configurations. Through full‐wave simulations and polarization‐resolved dark‐field spectroscopy of NW arrays, we demonstrate that hexagonal cross‐sections modify Mie‐mode properties: while degrading high‐index resonances, they enhance the NW‐substrate contact area, enabling more compact designs. The geometry transition from cylinders to hexagons introduces strong polarization‐dependent scattering effects, with measured spectral shifts between vertical and planar configurations exceeding 50 nm. These effects, together with GaP's intrinsic nonlinearity, provide unprecedented spectral tunability across the entire visible range, as validated by mode analysis correlating hexagonal NW resonances with classical Mie theory. Our results establish GaP NW as a versatile platform for tailored nanoantennas, with immediate applications in polarization‐sensitive light harvesting and nonlinear nanophotonics.

## 2 Results

### 2.1 NW Configurations and Their Optical Characteristics

Figure 1a illustrates the two studied configurations: (1) vertical NW arrays on Si and (2) planarized NW on quartz glass. The quartz substrate was chosen to enhance the optical contrast and optical action of the NW. For the following discussion, we introduce the excitation polarization notations (s‐/TM or p‐/TE) depicted in Figure 1a. The transverse dimensions of the NW hexagonal cross‐section are defined by the diameter d of the circumscribed circle. The following sections present a detailed comparative analysis of these configurations, examining how the transition from vertical (as‐grown) to planarized NW arrangements affects their optical behavior, with particular emphasis on polarization‐dependent Mie resonances and mode interactions in hexagonal nanostructures. The GaP NW investigated in this study were synthesized via molecular beam epitaxy (MBE, see details in the Methods), with as‐grown vertical structures on Si substrates shown in Figure 1b. NW can also be planarized on auxiliary substrates (e.g., quartz) through ultrasonication in liquid or mechanical transfer for optical characterization and device integration, as demonstrated by the representative SEM image in Figure 1c. Figure 1d shows a plane‐view SEM image revealing GaP NW hexagonal cross‐sections, with dimensions ranging from 60 to 200 nm, which enables diameter‐dependent optical response tuning.

**FIGURE 1:**
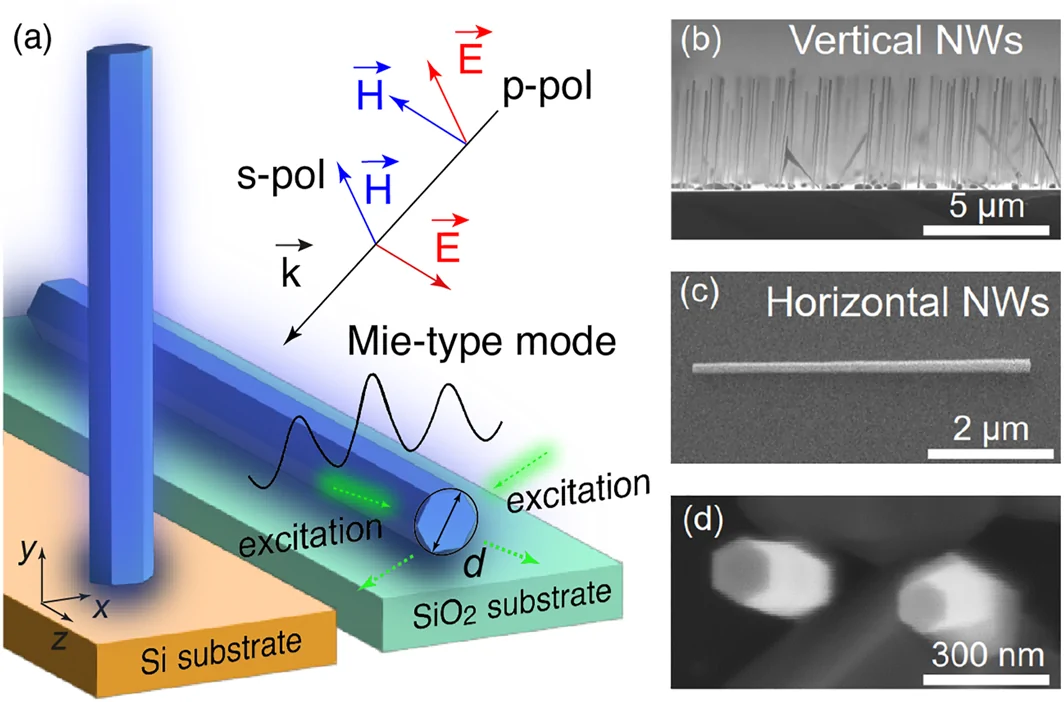
GaP epitaxial NW resonators. (a) Schematic view of a NW in vertical and horizontal (planarized) configurations with s‐ and p‐polarized excitation. SEM image of vertical (b) and horizontal (c) GaP NW; the size of NW is determined by d parameter. (d) Plane‐view SEM image depicting as‐grown NW hexagonal cross‐section.

The power of Mie theory stems from its analytical solutions for circular‐profile NW, which provide fundamental insights into system behavior, including geometric parameter dependencies and refractive index dispersion effects. While hexagonal structures support Mie‐type modes due to geometry close to cylindrical one, proper characterization requires comparative analysis of hexagonal and cylindrical structures, where the classical Mie modes are well‐defined.

For circular cross‐sections, there is an established classification of Mie resonances into electric and magnetic dipoles, quadrupoles, and higher‐order multipoles. The modes are manifested as pronounced features in the scattering cross‐section spectrum, with well‐known field patterns described by azimuthal and radial indices. By comparing the spectra and field profiles, we can match the modes of hexagonal rods with the Mie modes of circular cylinders. We should note that the structure is uniform along the z‐axis, which allows us to reduce the full‐wave 3D electromagnetic problem to a scalar 2D one without loss of generality. Consequently, we restrict our Mie mode characterization to the xy‐plane scattering profiles, enabling precise identification of resonant contributions to NW optical properties.

Using COMSOL Multiphysics Software, we simulated a 2D circular rod with the refractive index of GaP and a diameter of 140 nm, planarized on a glass substrate under plane‐wave excitation. The plane of incidence is orthogonal to the NW axis, and the angle of incidence is $65^{o}$. This angle corresponds to our dark‐field experimental setup, as will be discussed in the following. We used the dispersive complex refractive indices of GaP and glass from [41, 42] (see the corresponding data in Supporting Information S1: Figure S1). To study the optical illumination of the circular GaP rod, we calculated dimensionless scattering cross‐section (SCS) spectra normalized to the NW diameter, shown in Figure 2a,b by dashed curves for TE‐ (in‐plane electric field) and TM‐ (out‐plane electric field) polarized excitation. Note that for oblique incidence with respect to the NW axis, s‐ and p‐polarization notation is more suitable. In the case considered here, TE‐ and TM‐polarizations correspond to p‐ and s‐.

**FIGURE 2:**
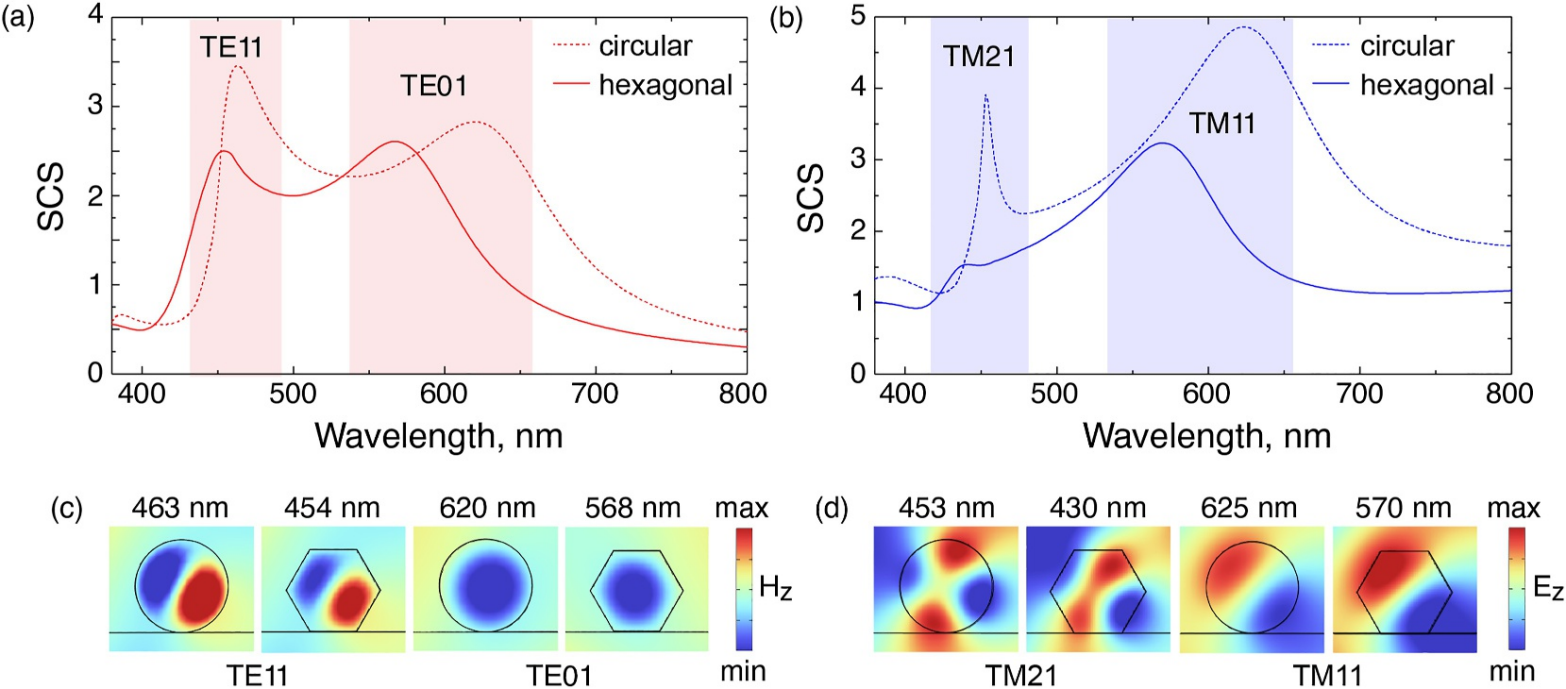
Dimensionless SCS spectra of the cylindrical and hexagonal horizontal GaP NW with d = 140 nm diameter under (a) TE‐ and (b) TM‐polarization. Scattering values are normalized to the geometrical cross section. Corresponding field patterns for z‐component of magnetic (c) and electric (d) field distributions over the NW cross‐section for TE and TM polarization respectively at the resonant wavelengths indicated at the top of each insert.

For TE polarization, a pair of features is observed: a broad Mie resonance with a long wavelength and a narrower one in the short‐wavelength range. A similar response is observed for TM polarization, but with the short‐wavelength resonance narrower than that in the TE spectrum. We also note that both narrow resonances appear as sharp asymmetric Fano lines with a dip at the shorter wavelength side.

The perturbation due to the substrate barely affects the Mie mode profiles. Thus, by analyzing the patterns of electric and magnetic field components along the z axis, we identify modes in the scattering spectra as TE11, TE21 and TM01, TM11, where the first index is azimuthal and the second index is radial. Remarkably, the frequency of the magnetic TE11 mode almost matches the frequency of the electric TM01 mode. The same is applicable for TE21 and TM11 modes [43]. Figure 2c,d show that for TE excitation, the field distributions in the NW cross‐section are oriented according to the plane wave angle of incidence and strongly localized inside the structure. In contrast, TM excitation leads to field distribution both inside the NW and in its vicinity.

Now we turn to the realistic hexagonal profile of an NW grown via MBE technique. The maximal diameter of this hexagon (referred to as size hereafter) is equal to the diameter d of the circumscribed circle. The scattering spectra of the hexagonal wires were compared with those of the circular rods. NW SCS in Figure 2a,b demonstrate similar behavior, but for hexagonal NW, SCS peaks have lower amplitudes and are slightly shifted to shorter wavelengths. These differences are likely due to the smaller area of the section of the hexagonal rod than the circular one. Note that the TE01 mode nearly preserves the scattering efficiency, whereas the TE11 mode decreases in amplitude. The narrowest TM21 Mie resonance broadens and significantly weakens. The main reason for this dramatic suppression of the TM21 scattering is the higher azimuthal index. Indeed, the higher the azimuthal index is, the more the mode resembles a whispering gallery mode. Thus, transforming circular NW into hexagonal ones leads to disruption of the field and its leakage into free space. The modes with a higher azimuthal index are suppressed even more strongly. Therefore, GaP NW fabricated with MBE are well‐suited for low‐index Mie modes, and for the visible range, the NW size should not be not very large.

We analyzed the resonant properties of the hexagonal NW considering the distribution of the electromagnetic field over its cross‐section (see Figure 2c,d). Electric and magnetic field distributions are very similar to each other and slightly modified compared with those of circular rods. Note that in the hexagonal NW, TM11 polarization enables stronger field confinement with the electric field hot spots at the NW‐substrate interface, which enhances the localization of the electric field and the radiation efficiency.

### 2.2 Scattering Cross‐Section and Electrical Energy Simulations for Different NW Sizes

As we mentioned above, the field distribution at the TM11 mode wavelength is characterized by two hot spots, with one located at the NW‐ substrate interface. To investigate how the NW geometry affects its scattering properties, we simulated SCS spectra of the NW with dimensions ranging from 100 to 140 nm, normalized to the NW geometrical cross section. The calculations were performed in the wavelength range of 380–800 nm for both TM and TE polarizations. The color maps in Figure 3a,b show the SCS spectra, with the Mie modes highlighted with white dashed curves. We note that in TM polarization, the intensity of the magnetic TM11 mode is dominant. Moreover, its intensity is the highest compared with the modes in TE polarizations. This means for illumination with unpolarized light, TM‐polarized mode will dominate, providing both strong field localization and high radiation losses.

**FIGURE 3:**
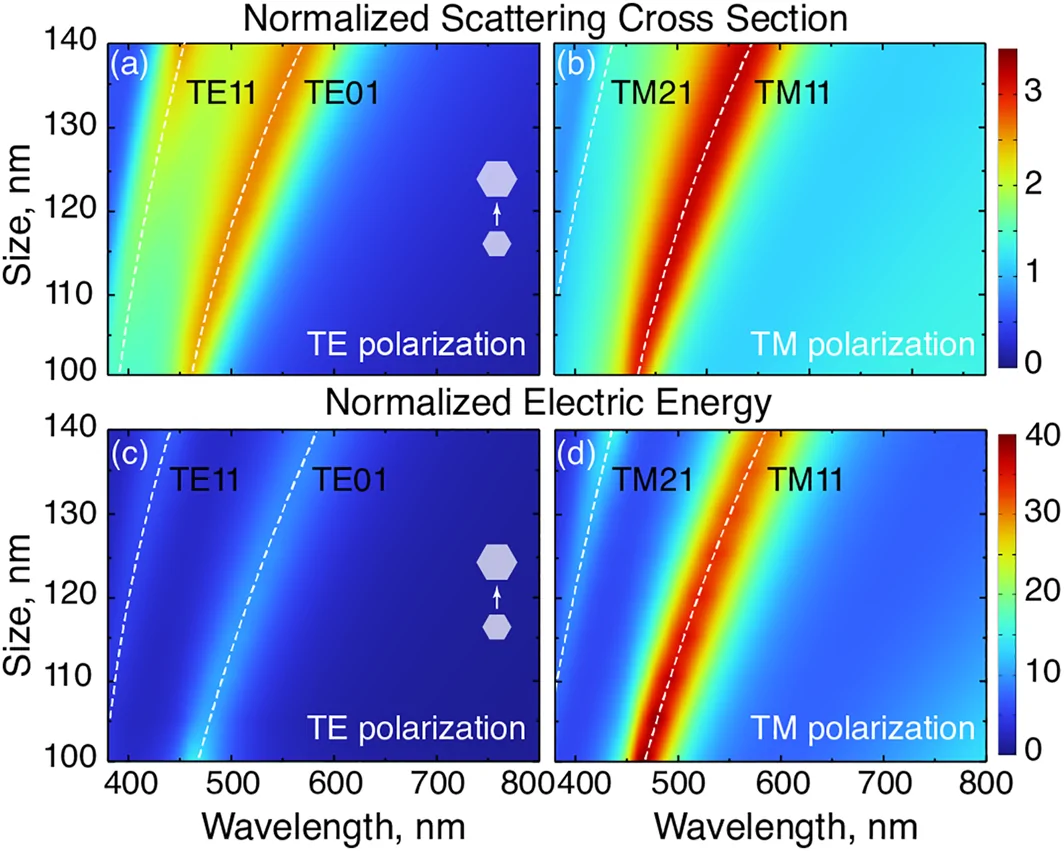
Calculated SCS spectra (top) and electric energy within the NW cross‐section (bottom) for (a, c) TE and (b, d) TM polarizations as a function of NW diameter. White dashed lines indicate the spectral Mie modes wavelength. Insets illustrate the NW size change. SCS spectra are normalized to the NW geometrical cross sections.

The spectral position of Mie modes of the NW is strongly influenced by the cross‐section size, and by the dispersion of the refractive index and absorption of GaP. However, in SCS spectra, the resonance peaks usually appear as Fano profiles with asymmetric lineshapes, and in general case, the mode wavelength does not match the maximum of the spectral feature, which complicates tracing the resonant wavelength of a Mie mode. In contrast, the dependence of energy accumulated in the NW on the wavelength exhibits a symmetric line in the scattering spectra. For this reason, we calculate the electric energy $\epsilon \epsilon_{0}\int |E{|}^{2}dS$ within the NW cross‐section. The simulation results for the electric energy normalized to the energy stored in the same volume of air for the system with no NW depending on the diameters for TE and TM polarizations are shown in Figure 3c,d. As the NW size increases, the intensity of Mie modes increases, and they shift to longer wavelengths. The TM11 mode is particularly prominent, exhibiting strong field localization inside the NW with efficient enhancement of the electric field.

### 2.3 Dark‐Field Spectroscopy and Far‐Field Simulation

Now, using the results of the theoretical analysis, we study Mie modes in GaP NW experimentally. Using dark‐field optical microscopy, we measured the characteristics of both horizontal and vertical configurations of the NW shown in Figure 1a,c. By analyzing the far‐field scattering spectra, we systematically investigate the wavelength‐dependent scattering behavior of an individual NW.

First, we investigated horizontally oriented individual NW planarized on a glass wafer. Several NW of gradually varying diameter in the range of 90–140 nm were chosen according to dark‐field optical images (see SEM visualization in Supporting Information S1: Figure S6). All measurements were performed using an objective x100 NA = 0.9, allowing intense excitation and efficient collection of the signal from an individual structure (Section 4 and Supporting Information S1: Figure S2). The calculated scattering of horizontal NW demonstrated a dependence on the evolution of the in‐plane angle upon excitation in both polarizations, see Supporting Information S1: Figure S3. The obtained scattering spectra of the studied NW were summed over the planar angle to simulate the all‐round irradiation. The resulting scattering spectra are normalized to their respective maximum values, which correspond to the peak intensity, and shown in Figure 4a. For clarity, the spectra are vertically offset. Each spectrum displays a maximum, shifting from 468 nm to 521 nm as the NW diameter increases. There is also a weak peak (marked by red arrows) at longer wavelengths, which similarly shifts from 459 nm to 600 nm.

**FIGURE 4:**
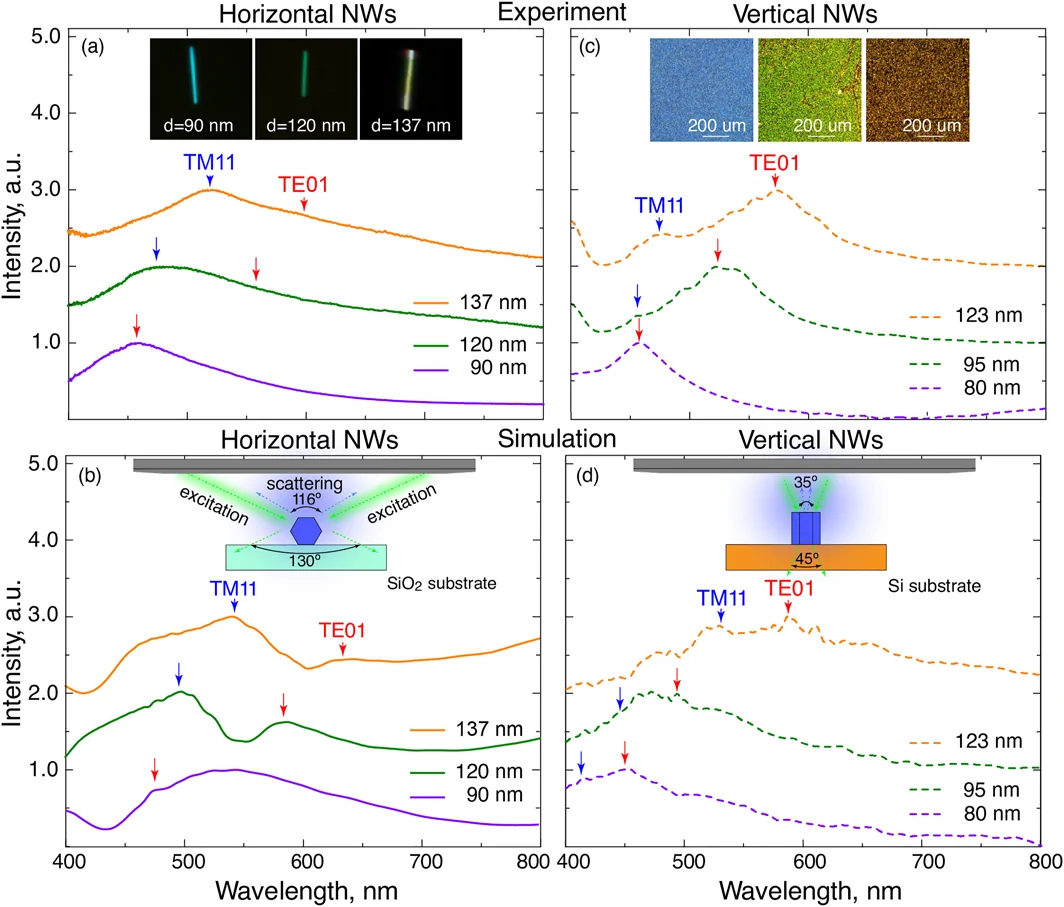
Experimental (top) and simulated (bottom) dark‐field spectra of GaP NW in horizontal (a, b) and vertical (c, d) for different NW diameters. The spectra are normalized to the eigenmaxima and shifted along the vertical axis. Insets in the top panels show GaP NW dark‐field optical microscopy images. Insets in the bottom panels show illumination and signal collection in the dark‐field scheme. Red arrows indicate TE01 modes, blue arrows indicate TM11 modes.

Second, we turn to the vertical configuration. Vertically aligned NW arrays on the growth Si substrate enable simultaneous excitation of multiple nanostructures due to their high density. Therefore, a low‐magnification 10x objective, providing a larger excitation spot, was employed. In this case, the incident angle is $22.5^{o}$. Scattering spectra were acquired from three NW arrays with different diameters. Dark‐field optical microscopy revealed a clear color contrast, with the NW appearing green and yellow in different samples (see insets in the Figure 4c). To determine their diameters, we used SEM imaging, yielding average values of 80 nm (blue region), 95 nm (green region), and 123 nm (yellow region). The corresponding scattering spectra (Figure 4c) exhibited distinct peaks at 463 nm (blue), 529 nm (green), and 575 nm (yellow), confirming diameter‐dependent resonant scattering.

Although the experimental spectra show several peaks, their interpretation requires an additional set of simulations taking into account the actual geometry. The scattering cross‐section measurements were simulated using CST Microwave Studio. We assumed that the distance between NW is large enough for the coupling effects to be neglected [44]. We simulate a single NW 3 μ m and 7 μ m in longitudinal size (for the horizontal and vertical configurations). The dependence of the scattering intensity spectra on the NW length is discussed in Supporting Information S1: Figure S5. The sample is illuminated by a plane wave incident with an axial angle of $65^{o}$ (horizontal) or $22.5^{o}$ (vertical) and variable azimuthal angle, which simulate the dark‐field objective action. The intensity of light scattered by the NW into the objective NA is integrated. Note that for each azimuthal angle, the illumination is either s‐ or p‐polarized, defined by the plane of incidence. However, the NW modes are denoted as TE and TM, described above.

Figure 4b demonstrates the simulation results for horizontal NW with size s *d* = 90, 120, and 137 nm, and Figure 4d, for vertical NW with sizes *d* = 80, 105, and 123 nm, shown by dashed curves. We observe several spectral features corresponding to Mie‐like modes in both NW configurations. Based on the field distributions in NW, we distinguished the TM11 and TE01 modes in both configurations. The wavelength of TE01 is about 80 nm longer than that of TM11. Moreover, in the spectra, all modes are shifted to longer wavelengths as the NW diameter increases. This long‐wavelength shift of Mie mode position originates from the increase of the optical path length and effective mode volume inside larger NW. Electromagnetic modes supported over a larger spatial extent lead to larger wavelengths. In addition, the electromagnetic hot spots oriented along the angle of incidence become oriented into the NW, so that the localized electric field at the NW‐substrate boundary is enhanced (see Supporting Information S1: Figure S3).

The spectra shown in Figure 4 reveal a good agreement between the experimental measurements and theoretical simulations for both horizontal and vertical NW configurations. Experimental spectra comprise two peak features (marked with arrows in Figure 4a,c), whose positions shift with the NW size, similar to the simulations. We attribute them to the spectral features of TM11 and TE01 in accordance with the theoretical analysis. For the horizontal NW, the TM11 peak is more pronounced, whereas the TE01 peak remains broad and weakly distinguished in the spectra. The simulated spectra of both NW configurations contain low‐intensity fringes and strong TM11 and TE01 modes, which we successfully identified in the experimental spectra. We notice that the measured spectra are slightly shifted relative to the calculated ones, so in the vertical configuration, we do not observe the TM11 mode for the small NW (d = 80 nm in vertical and d = 90 nm in horizontal configuration) in the measured wavelength range. The possible reason for this spectral shift is the surface oxidation layer changing the efficient NW diameter. In addition, both experimental and theoretical spectra show different peak intensity patterns for horizontal and vertical configurations. For the horizontal configuration, the TM11 peak has a higher intensity than the TE01 one, whereas for the vertical one, the situation is the opposite. The reason is the direction of illumination, which more efficiently excites the TM11 mode for the horizontal configuration and the TE01 mode for the vertical configuration.

## 3 Discussion and Conclusions

Now we discuss the features related to the hexagonal shape of GaP NW. Compared with cylindrical ones, hexagonal facets provide larger contact area, improving mechanical stability, heat dissipation, and electrical interaction. Notably, the TM11 hotspot is located at the NW–substrate interface, providing a robust mechanism for near‐field energy concentration and efficient light–matter coupling. This principle is directly applicable to biosensing, where strong field localization enhances fluorescence from labeled biomolecules. Topologically optimized infinity‐shaped GaP nanoantennas demonstrated fluorescence enhancement up to two orders of magnitude [45]. Although hexagonal NW are simpler, scalable epitaxial growth enables cost‐effective large‐area biosensor chips. Importantly, scattering maxima do not coincide with energy maxima due to Fano interference, which must be considered in device design.

The hexagonal shape limits the number of supported Mie modes. Higher‐order modes resemble whispering gallery modes and leak due to deviation from circular symmetry; however, low‐order modes dominate practical applications. Few studies rely on modes above quadrupoles, and in the visible range, low‐order resonances match NW diameters of 80–200 nm. While ultra‐high‐Q resonances such as quasi‐BIC are attractive, they are highly sensitive to imperfections [46]. Bottom‐up growth introduces dimensional variations, making ultra‐high‐Q platforms impractical. In contrast, hexagonal NW favor robust low‐order Mie modes with predictable, broadband response, suitable for scalable nanophotonic and quantum optical devices that do not require thick NW.

The results highlight GaP as a strong platform for visible‐range nanophotonics and nonlinear optics. Unlike silicon, which suffers from absorption in the visible range, GaP combines broad transparency with high refractive index for tight confinement [47]. Its non‐centrosymmetric structure provides intrinsic second‐order nonlinearity, enabling SHG and electro‐optic effects absent in Si. Although GaAs offers higher nonlinearity, its narrower bandgap limits it to IR [47]. Lithium niobate has a lower refractive index and requires complex integration [48]. Thus, dense epitaxial GaP NW arrays can act as scalable nonlinear metasurfaces, comparable to top‐down GaP metasurfaces with a high SHG efficiency [49]. Near‐perfect lattice matching to Si further enables monolithic integration [47], and thus, GaP perfectly combines the III–V performance and CMOS scalability.

In summary, we theoretically and experimentally characterized Mie modes in self‐assembled GaP NW grown by MBE in vertical and horizontal configurations. The hexagonal cross‐section modifies Mie‐type modes: higher‐order modes (e.g., TM21) are suppressed, whereas low‐order modes (TE01, TM11) remain dominant with spectral shifts. Simulations and dark‐field spectroscopy confirm strong dependence of the mode features on the NW diameter, incident polarization, and substrate interaction. TM11 mode is strongly localized at the NW–substrate interface, enhancing light–matter interaction. The hexagonal geometry also improves mechanical and thermal properties and supports robust fabrication‐tolerant resonances. Combined with GaP's wide bandgap, high refractive index, intrinsic second‐order nonlinearity, and lattice compatibility with Si, these NW provide a versatile, scalable platform for sensing, nonlinear metasurfaces, and integrated photonic and quantum devices.

## 4 Methods

### 4.1 NW Fabrication

Self‐catalyzed gallium phosphide nanowires (GaP NW) were grown using a Veeco GEN III molecular beam epitaxy (MBE) system. The nanostructures were formed on Si (111) substrates via a self‐catalyzed vapor‐liquid‐solid (VLS) mechanism, employing solid‐source MBE. A valved cracker cell supplied the $P_{2}$ molecular beam. Prior to growth, the Si substrates underwent the Shiraki cleaning method [50], concluding with wet chemical oxidation in a base piranha solution (a 1:1:3 mixture of ammonia, hydrogen peroxide, and water) [51]. To facilitate nanowire formation, the oxidized Si (111) substrates were thermally annealed in the MBE chamber at 760°C for 30 min. This step promoted the creation of pinholes in the surface oxide layer, which act as nucleation sites for catalytic gallium droplets. The fluxes of group‐III and group‐V elements were monitored using a Bayard‐Alpert vacuum gauge, measuring their beam equivalent pressures (BEP). The Ga‐cell BEP was set at $8\times 10^{-}8$ Torr, corresponding to a GaP planar growth rate of 3.17 nm/min (190 nm/h), and remained constant during the NW growth. NW formation began with the simultaneous opening of the Ga and $P_{2}$ shutters. To fully consume the catalytic gallium droplets, the samples were exposed to a $P_{2}$ flux during the cooling phase. In situ reflection high‐energy electron diffraction (RHEED) analysis revealed that the NW grow vertically along the Si [111] direction, retaining the zinc‐blende (ZB) crystal structure characteristic of bulk GaP. To control the lateral dimensions of the nanowires, Ga droplets of varying diameters were employed. A comprehensive description of the detailed procedure and study on self‐catalytic GaP NW growth on Si (111) can be found in a previous publication [52].

Examination of NW described elsewhere [53] reveals a high density of rotational twins along the < 111> growth axis, characteristic of the self‐catalyzed vapor‐liquid‐solid method for III‐V materials. Critically, these defects occur within the ZB lattice; we do not observe the formation of extended, pure wurtzite segments [54, 55]. Therefore, the optical properties of our NW are governed by the electronic structure of bulk ZB GaP, justifying the use of the isotropic, bulk ZB dielectric function for modeling. While introducing planar defects, the twinning does not fundamentally change the phase or the relevant optical constants across the visible spectrum studied here.

### 4.2 Experimental Measurements

Dark‐field setup was based on an optical microscope Soptop RX50M modified with an additional optical channel connected to a spectrometer (Optosky ATP5020P) via fiber. Measurements were carried out in reflection geometry via dark‐field objectives (x10 NA = 0.3 for vertical NW and x100 NA = 0.9 for horizontal NW, both objectives from Nikon MPlan Fluor series) from the round spot with a diameter of 10 microns following the schematic in [56]. Samples were illuminated by a halogen lamp.

After that, for each individual NW, we measured a set of four dark‐field spectra under the same conditions to evaluate the scattering intensity: (1) spectrum from the NW—“sample”, (2) spectrum from the bare substrate ‐ “background”, (3) spectrum of the Spectralon diffuse reflectance standard (Ocean Insights WS‐1‐SL)—“reference”, (4) spectrum with the illumination source turned off—“dark”. The resulting scattering spectra were calculated via the following expression:

(1) $$I_{\mathrm{scattering}}=\frac{\left(I_{\mathrm{sample}}-I_{\mathrm{background}}\right)}{\left(I_{\mathrm{reference}}-I_{\mathrm{dark}}\right)}$$

All the measured intensities were normalized to the signal accumulation time. The signal intensity was found to linearly depend on the accumulation time for our time range.

## Author Contributions

**Aleksandra A. Kutuzova:** visualization, writing – original draft, investigation, formal analysis, conceptualization, data curation. **Alexey Kuznetsov:** conceptualization, investigation, data curation, writing – original draft, visualization. **Valeriy M. Kondratev:** resources. **Vladimir V. Fedorov:** resources. **Ivan S. Mukhin:** resources. **Aleksandr S. Slavich:** resources. **Aleksey V. Arsenin:** resources. **Alexey D. Bolshakov:** conceptualization, writing – review and editing, project administration, funding acquisition. **Mikhail V. Rybin:** conceptualization, writing – review and editing, supervision, project administration, funding acquisition.

## Funding

A.K. acknowledges Russian Science Foundation (Grant 25‐12‐00135) for support of the experimental work. A.V.A. thanks the Ministry of Science and Higher Education of the Russian Federation (project FSMG‐2025‐0005) for support of the paper preparation. V.M.K. thanks the Ministry of Science and Higher Education of the Russian Federation (project FSRM‐2026‐0004) for support of the SEM imaging. A.D.B. acknowledges Saint‐Petersburg State University for a research project 122040800254‐4 supporting the data analysis. M.V.R acknowledges support from the Russian Science Foundation (Grant 25‐12‐00213).

## Supporting information


Supporting Information S1

## Data Availability

The data that support the findings of this study are available from the corresponding author upon reasonable request.
