# Supplementary material for: Harnessing Interfacial Field Localization in Hexagonal GaP Mie Resonators
Source: Nanophotonics. 2026 Apr 30;15(9):e70085. doi: 10.1002/nap2.70085 (PMC13642546; doi:10.1002/nap2.70085)
Supplement: Supplementary file 1 [file NAP2-15-e70085-s001.pdf]

Supporting Information for

Harnessing Interfacial Field Localization in Hexagonal GaP Mie Resonators

Aleksandra A. Kutuzova[1], Alexey Kuznetsov[2,3], Valeriy M. Kondratev[2,3],
Vladimir V. Fedorov[2,5], Ivan S. Mukhin[2,5], Aleksandr S. Slavich[3],
Aleksey V. Arsenin[3], Alexey D. Bolshakov[2,3,4], and Mikhail V. Rybin[*,1,6]

[1]School of Physics and Engineering, ITMO University, St. Petersburg 197101, Russia
[2]Alferov University, Khlopina 8/3, 194021 Saint Petersburg, Russia
[3]Moscow Center for Advanced Studies, Kulakova str. 20, Moscow, 123592 Russia
[4]Faculty of Physics, St. Petersburg State University, Universitetskaya Emb. 7-9, St. Petersburg, 199034 Russia
[5]Peter the Great Saint Petersburg Polytechnic University, Politekhnicheskaya 29, Saint Petersburg, 195251 Russia
[6]Ioffe Institute, St. Petersburg 194021, Russia

E-mail: m.rybin@metalab.ifmo.ru

Section 1 – Refractive and absorption indices
Section 2 – Experimental Section
Section 3 – Far-field scattering simulation
Section 4 – Planar angle evolution
Section 5 – Field distributions
Section 6 – Longitudinal size influence
Section 7 – SEM visualization
Section 8 – Scattering comparison
Section 9 – Error bars

1. Refractive and absorption indices

In figure S1 we demonstrate refractive and absorption indices used for calculations by COMSOL Multiphysics.

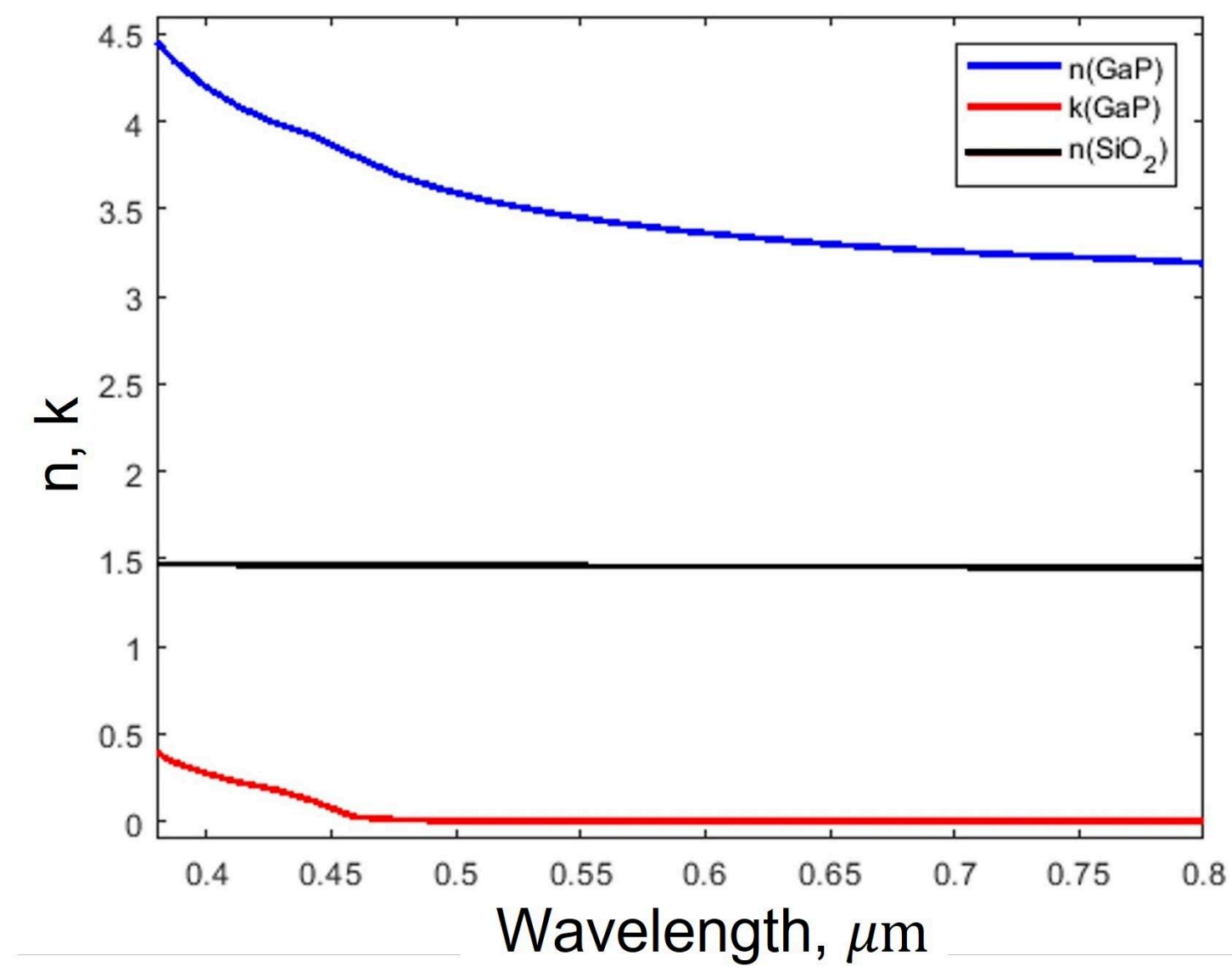

Figure S1: Refractive indices n, k of GaP and SiO2. The data from Refs. [1,2].

2. Experimental Section

Top view of figure S2: the excitation channel through which the excitation light (green arrows) propagates is designated as input. The electric field vector is oriented in accordance with planar angle φ_i in s and p polarizations. Also, we determine the TM polarization, where the electric field vector is directed along the NW, and TE polarization, respectively, across. NW is shown schematically not to the scale of the system. The output channel collects scattered light (blue arrows) to the objective back.

Side view. The light excites the NW at an azimuthal angle $2\theta_i = 130^o$, where θ_i is relative to the normal. Scattered light is collected at an angle $2\theta_{NA} = 116^o$ corresponding to the numerical aperture of the objective, where θ_{NA} is relative to the normal. The illumination is done at a wide angle to avoid specularly reflected light entering the objective.The experimental scheme is presented for the horizontal configuration of NW; for the vertical one, the same scheme was used, but with an objective aperture of 0.3 and $2\theta_{NA} = 35^o$.

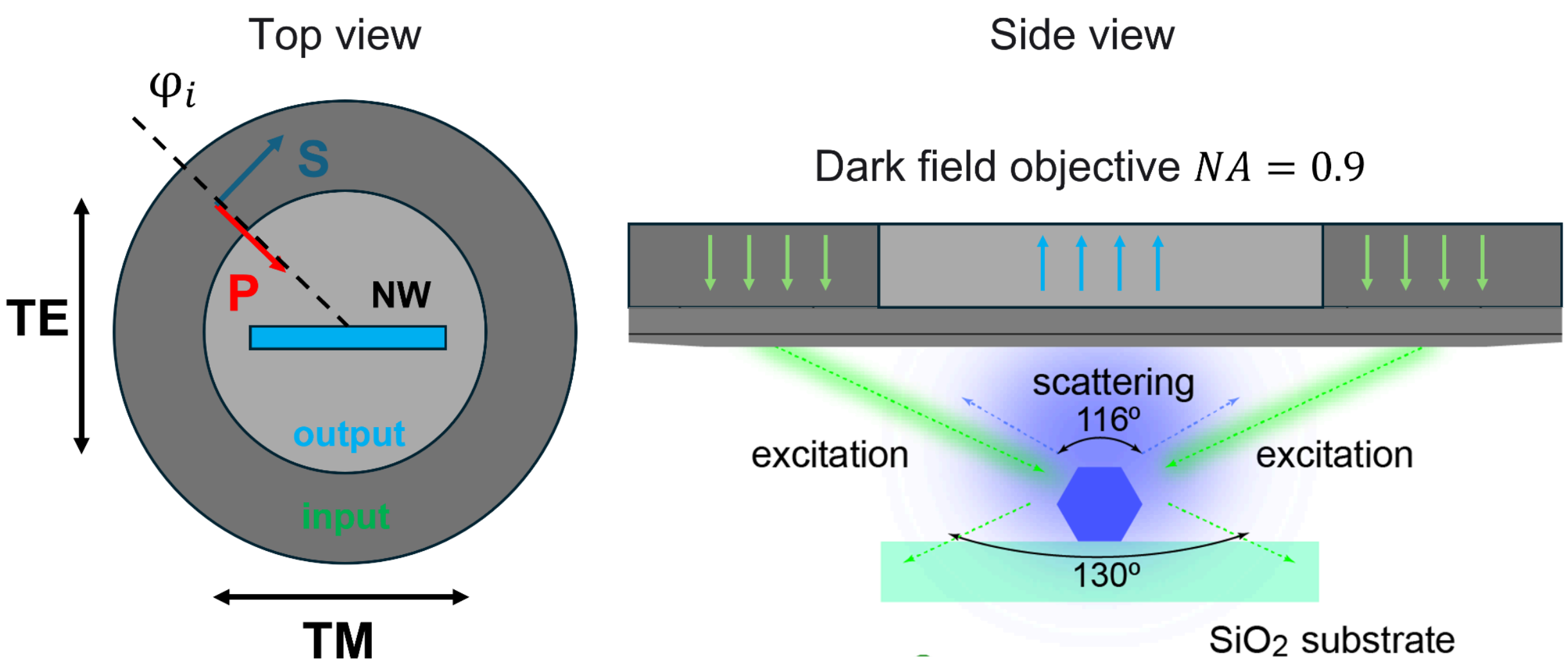

Figure S2: Scheme of the experiment measurements.

3. Far-field scattering simulation

In the simulated systems, that reproduce experimental measurements (dark field measurement scheme is presented in fig.S2), the sample is illuminated from the input lens by an axial angle of the incident wave θ_i from the normal and by planar angle φ_i. Also, light is scattered by the NW into output objective at angles φ_s and $\theta_s = \theta_{NA}$ corresponding to the numerical aperture of the objective. To calculate intensity of scattered light in s polarization I_S for planar angles φ_i we perform equation:

$$I_s(\theta_i, \varphi_i) = \int_0^{\theta_{NA}} \int_0^{2\pi} I_s(\theta_s, \theta_i, \varphi_s, \varphi_i) d\theta_s d\varphi_s \qquad (1)$$

where $\theta_{NA} = 58^o$, $\theta_i = 65^o$. Planar angle φ_i was taken with a 15 degrees step in range from 0 to 90. For scattered light in p polarization respectively:

$$I_p(\theta_i, \varphi_i) = \int_0^{\theta_{NA}} \int_0^{2\pi} I_p(\theta_s, \theta_i, \varphi_s, \varphi_i) d\theta_s d\varphi_s \qquad (2)$$

Next, we calculate scattering intensities in TE $I_{TE}(\theta_i)$ and TM $I_{TM}(\theta_i)$ polarizations using $I_P(\theta_i,\varphi_i)$ and $I_S(\theta_i,\varphi_i)$ restoring their projections relative to φ_i angle:

$$I_{TE}(\theta_i) = \int_0^{\pi/2} (I_s(\theta_i, \varphi_i) sin(\varphi_i) + I_p(\theta_i, \varphi_i) cos(\varphi_i)) d\varphi_i \qquad (3)$$

$$I_{TM}(\theta_i) = \int_0^{\pi/2} (I_p(\theta_i, \varphi_i) sin(\varphi_i) + I_s(\theta_i, \varphi_i) cos(\varphi_i)) d\varphi_i \qquad (4)$$

where integration limits from 0 to $\pi/2$ allow scattering in a 2π. Total SCS intensity was carried out as half sum of $I_{TE}(\theta_i)$ and $I_{TM}(\theta_i)$ and calculated for both horizontal and vertical NWs: $(I_{TE} + I_{TM})/2$.

4. Planar angle evolution

During the simulation of the far-field scattering intensity of the nanowire, we accounted for the rotation of the scattering angle φ, which resulted in a change in the shape of the resulting spectra in both polarizations. In figure S3 at the normal angle of incidence φ_i for p-polarized irradiation we observe dominant TE01 mode that shifts to the short-wave region and demonstrates higher intensity with increasing of NW size from 120 to 137 nm, wherever intensity of TE11 mode is depressed at the initial state. During the φ_i evolution the scattering intensity of TE01 mode diminishing, while TE11 starts to gradually grow especially for larger sizes. Also, there is smooth arising of TM11 mode, the intensity of which reaches its maximum at phi=90, which corresponds to the oscillation of the electric field along the NW. For polarization we observe TM11 mode that shifts to the short wavelength region and demonstrates higher intensity with increasing of NW size. The scattering intensity of TM11 mode decreases with φ_i, while TE01 mode appears. In each polarization, when planar angle of irradiation is rotated by 90 degrees, orthogonal polarization modes are excited and contribute to the resulting scattering of the NW.

.

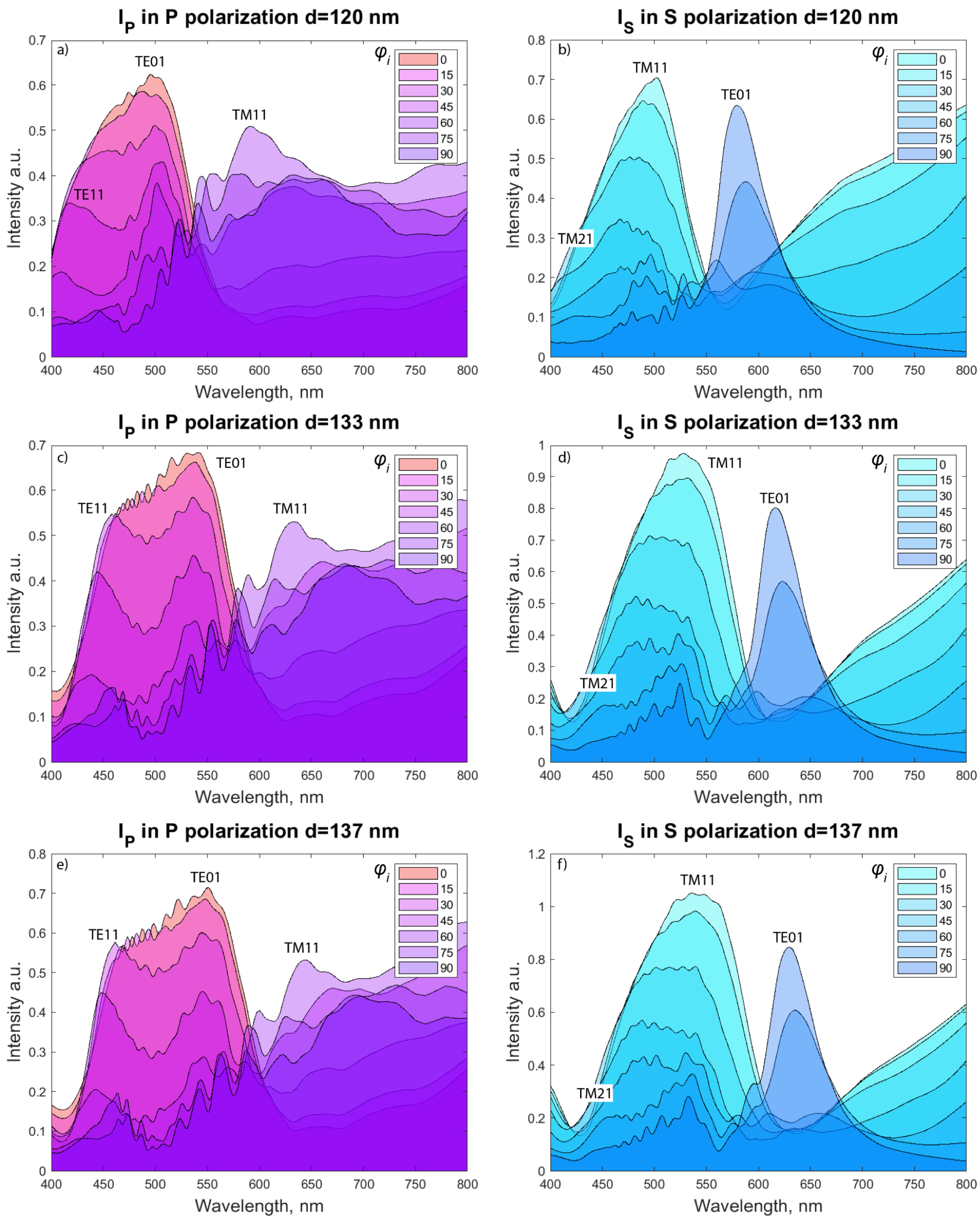

Figure S3: a), c), e) - Scattering intensity in p polarization with planar angle φ_i evolution from 0° to 90° in a wavelength range from 400 to 800 nm form *d*=120, 133 and 137 nm respectively. b), d), f) - Scattering intensity in s polarization with planar angle φ_i evolution from 0° to 90° in a wavelength range from 400 to 800 nm form *d*=120, 133 and 137 nm respectively.

5. Field distributions

To analyze the features in the scattering intensity spectra of horizontal and vertical NW configurations, we consider the contributions of the scattering intensities in the two polarizations separately in the 400-800 wavelength range. In Figure S4a, the red curves show the scattering intensities in the TE polarization, and the blue curves show the scattering intensities in the TM polarization for nanowires with diameters of 90, 120, and 137 nm. The spectra exhibit maxima, indicated by blue and red arrows corresponding to the dominant polarization. The insets show the electric Ex and magnetic Hx field distributions of modes at the corresponding wavelengths. The field distributions are oriented in accordance with the angle of incidence oblique to the surface and coincide with the TM and TE profiles of the Mie-modes.
In Figure S4b we demonstrate the scattering intensities on vertical NW of 80, 95, 123 nm. Here the electric and magnetic fields in TM and TE modes are shown in the xy plane.

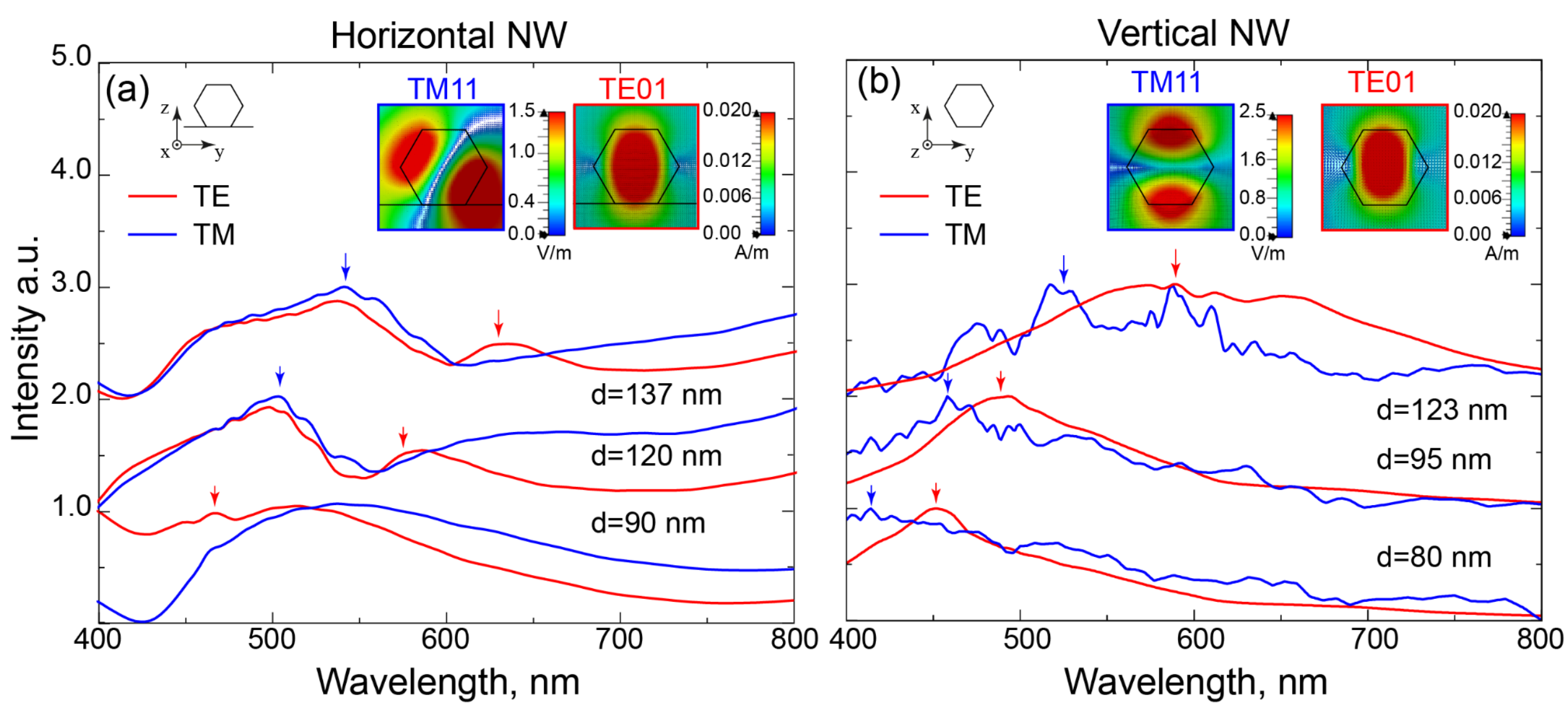

Figure S4: Scattering intensity of both horizontal and vertical NW with TM11 and TE01 Mie-modes distributions.

6. Longitudinal size influence
The model uses open boundary conditions. Nanowire lengths of 3 μm (horizontal orientation) and 7 μm (vertical orientation) were chosen as optimal for the calculations. In Figure S5a, we show the scattering intensity of vertical NW with a cross-section dimension of 123 nm and transverse dimensions of 3, 5, and 7 μm. Since the excitation angle was 22.5 degrees from the normal, Fabry-Perot peaks are excited in the polarization where the projection of the electric field onto the growth axis of the nanowire is maximum (corresponding to the s polarization of the full and TM polarizations relative to the NW). At 3 μm, they are strongly pronounced and smooth out when the longitudinal size increases to 7 μm. In Figure S5b we show the scattering intensity of horizontal NW with a cross-section dimension of 137 nm and transverse dimensions of 1 and

3 µm and observe fringes when excited by n-polarized light with the E field oriented across the NW.

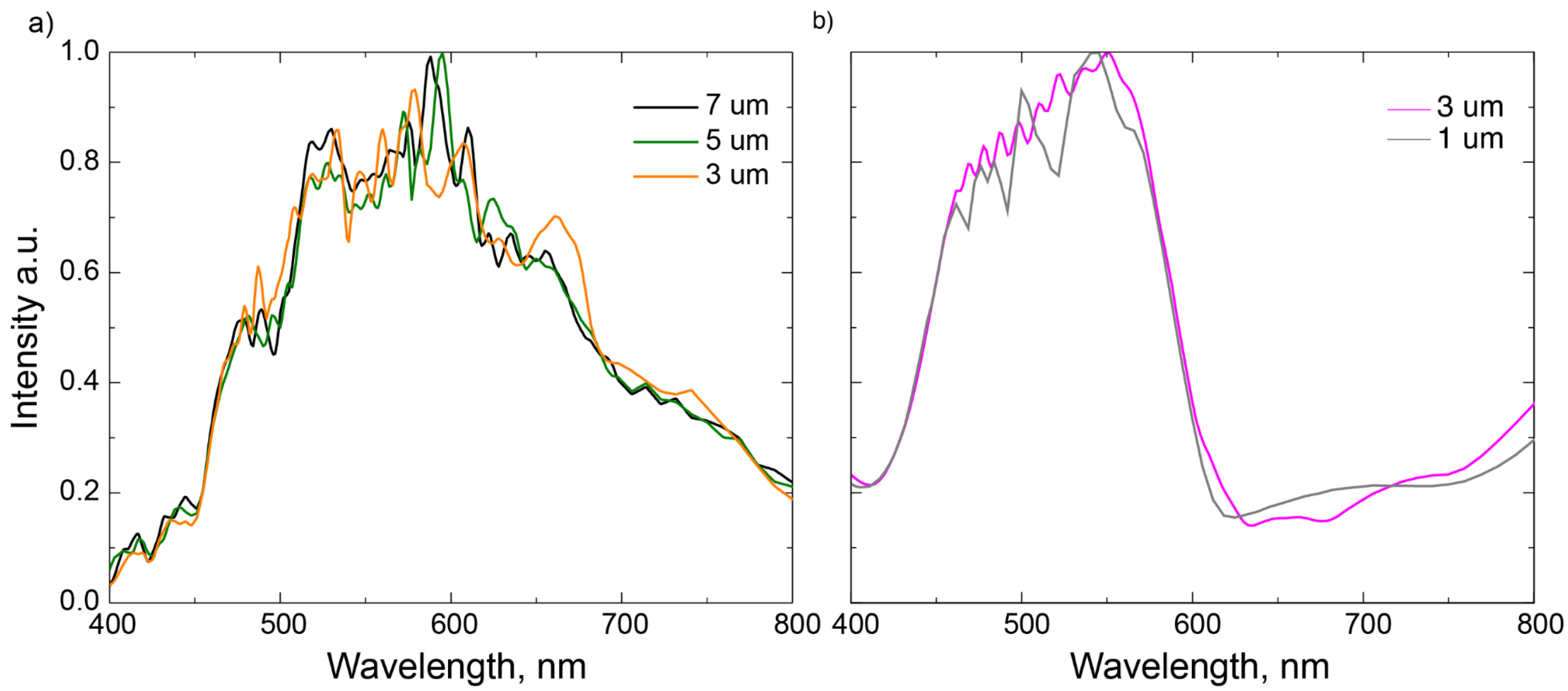

Figure S5: Scattering intensity of vertical NWs with longitudinal sizes. a) Vertical configuration of NW with d=123 nm, black, green and orange curves correspond to 3, 5, 7 µm respectively. b) Horizontal configuration of NW with d=137 nm, P polarization, gray, pink curves correspond to 1, 3 µm respectively. The data are normalized to the maxima.

7. SEM visualization

For demonstration of both horizontal and vertical individual GaP NW size characterization the additional measurements were provided. NWs geometry and dimensions were evaluated via SEM procedure in Figure S6.

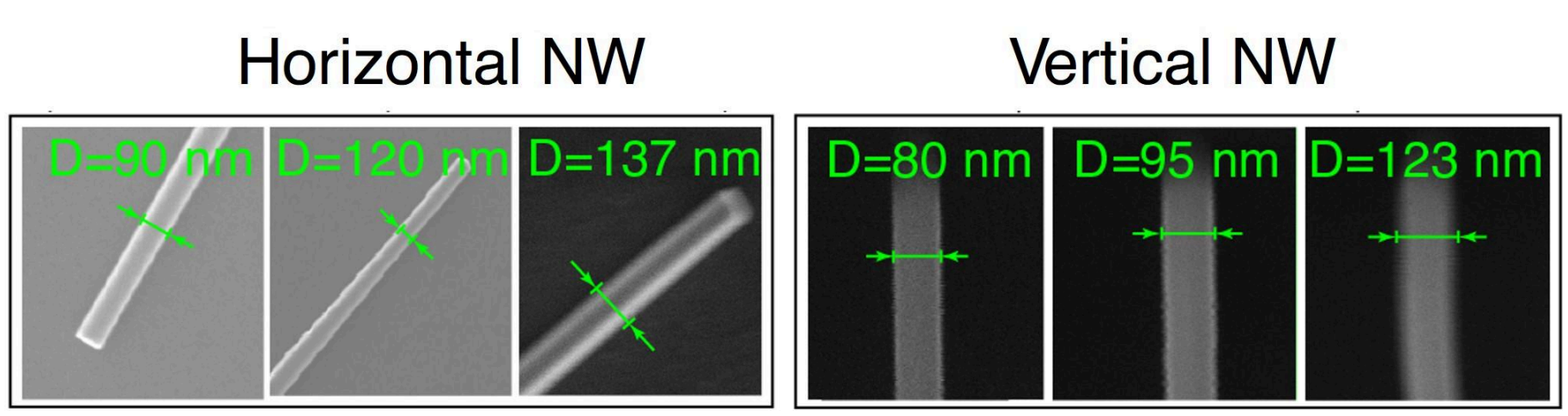

Figure S6. SEM s of GaP NWs of different diameters.

8. Scattering comparison

The intensity of the detected scattered light is determined both by the collection geometry and by the radiation pattern of the nanowire. A larger numerical aperture allows the objective to integrate the emitted radiation over a wider solid angle, which directly increases the measured signal. In addition, the orientation of the nanowire defines the spatial distribution of the scattered field. A horizontally oriented nanowire radiates a significant fraction of its energy toward the direction normal to the substrate, whereas for a vertically oriented nanowire the radiation

maximum is shifted toward lateral directions. As a result, in a top-collection configuration the horizontal geometry enables more efficient collection of scattered light. A further contribution arises from the interaction with the substrate, which redistributes the angular distribution of the emitted power differently for the two orientations. The scattering intensity of horizontal and vertically oriented nanowires shown in Figure S7. The simulation was performed for two nanowires of the same diameter d=120 nm, and the signal collection angle corresponds to the objective apertures. As can be seen, the horizontal NW (pink dotted curve) scatters about 5 times more intensively than the vertical NW (green curve).

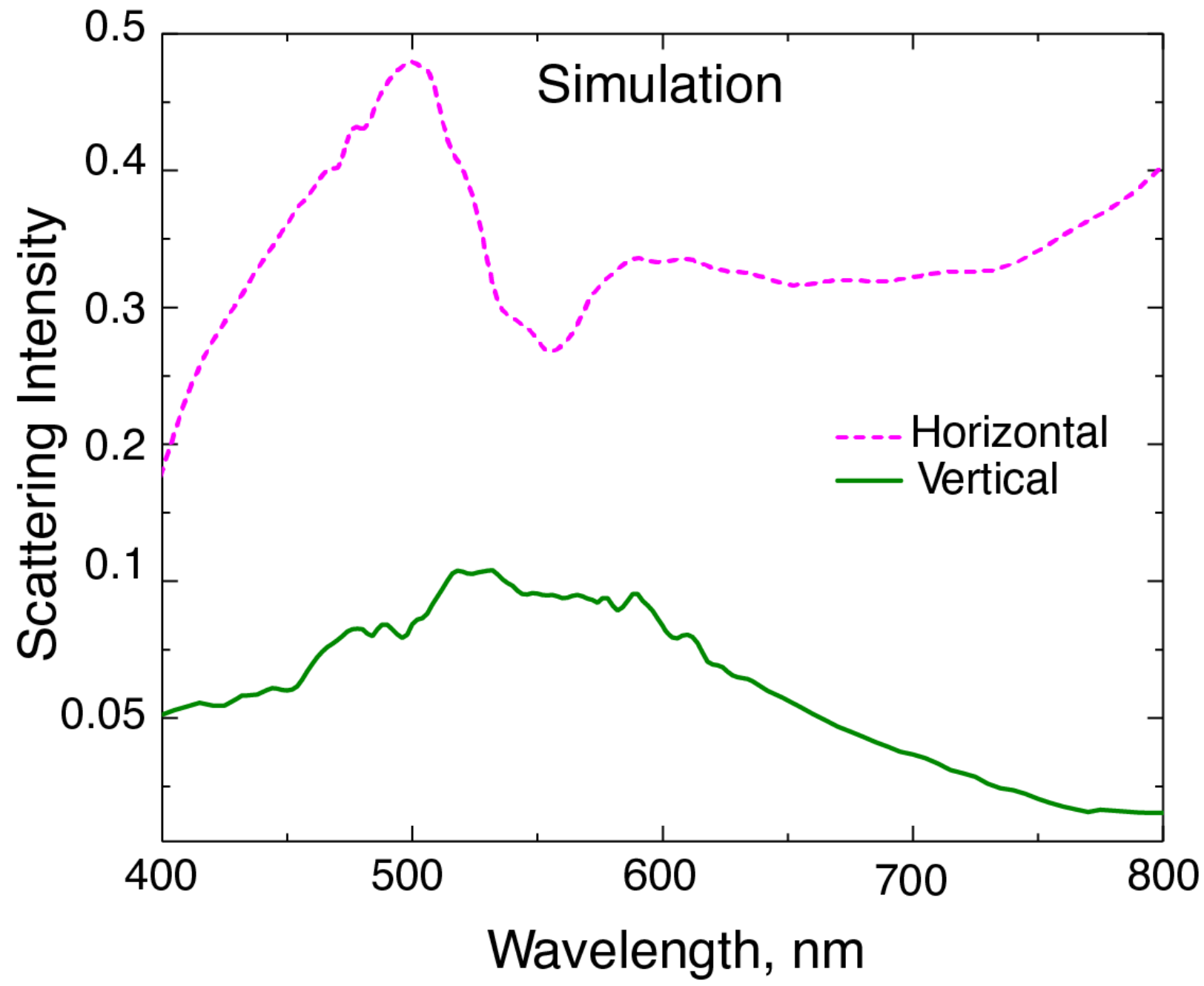

Figure S7. Comparison of scattered Intensity from horizontal and vertically oriented NW.

9. Error bars

Experimentally measured spectra were averaged and standard error was calculated for over 10 NWs of the same diameter and 10 spots on each array area (see Fig.S8). The calculated standard error does not exceed 2.7%. But, maximum error for arrays (Fig.S8d and c) ~2 times higher than for single NWs due to the statistical variations of the NWs diameter over the dark-field spectra collection spot.

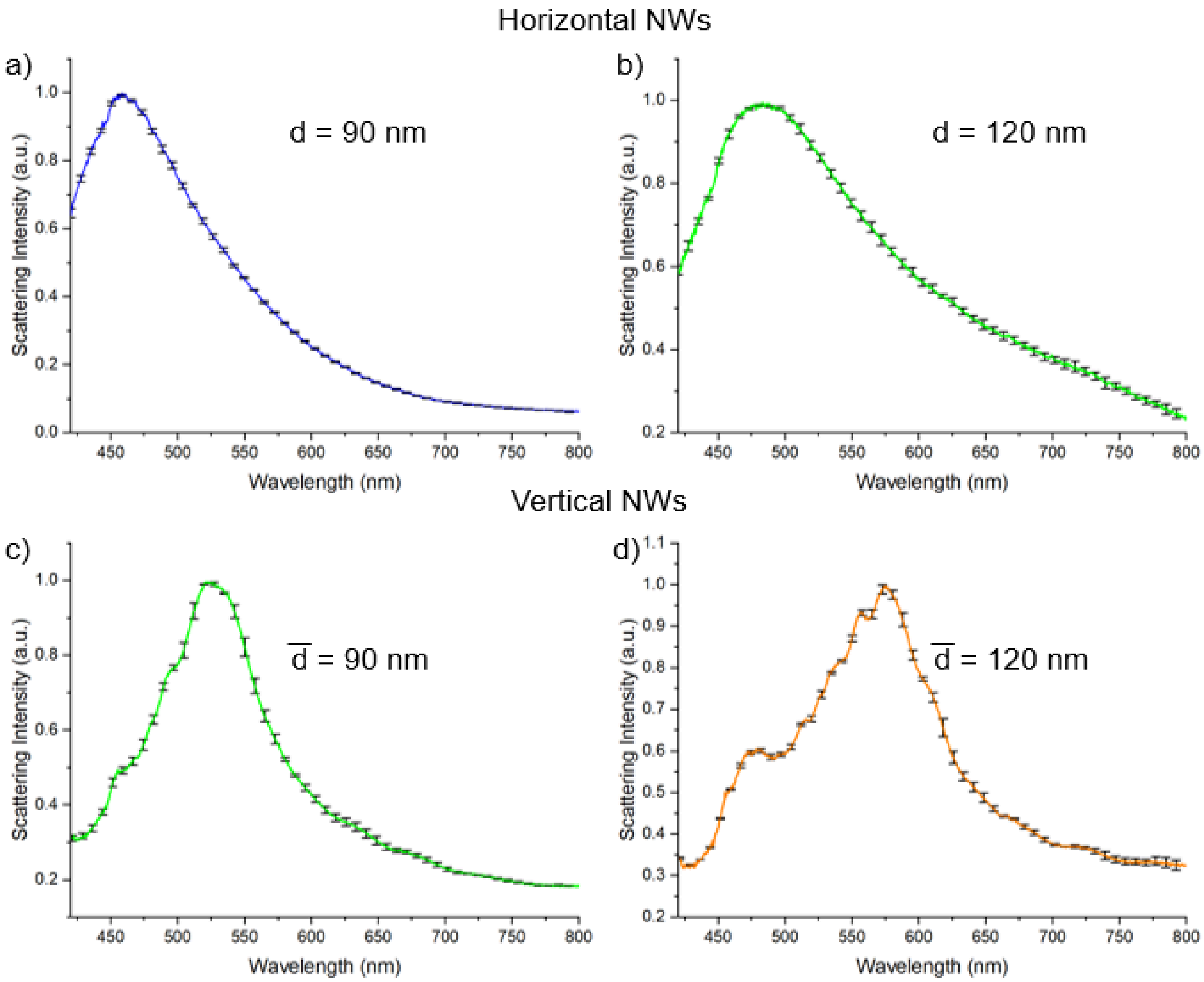

Figure S8. Experimental results with error bars.

References

[1] Aspnes, D. E., & Studna, A. A. (1983). Dielectric functions and optical parameters of si, ge, gap, gaas, gasb, inp, inas, and insb from 1.5 to 6.0 ev. *Physical review B*, *27*(2), 985.

[2] Malitson, I. H. (1965). Interspecimen comparison of the refractive index of fused silica. *Journal of the optical society of America*, *55*(10), 1205-1209.
